# The Longitudinal Physiological Reference Values of Middle Cerebral Artery Blood Flow Velocity in Extremely Preterm Infants: A Study Utilizing Multimodal Cerebral Hemodynamic Monitors

**DOI:** 10.3390/children13091135

**Published:** 2026-08-25

**Authors:** Wei-Hung Wu, Yu-Tang Juan, Shu-Yu Lin, Ming-Chou Chiang, Mei-Yin Lai, I-Hsyuan Wu, Shih-Ming Chu, Reyin Lien, Kai-Hsiang Hsu

**Affiliations:** 1 Division of Neonatology, Department of Pediatrics, Chang Gung Memorial Hospital Linkou Branch, Taoyuan 33305, Taiwan; ch60819@cgmh.org.tw (W.-H.W.); yoyodiy@cgmh.org.tw (Y.-T.J.); newborntw@gmail.com (M.-C.C.); a9275@cgmh.org.tw (M.-Y.L.);; 2 School of Medicine, Chang Gung University, Taoyuan 33302, Taiwan; 3 Department of Pediatrics, Chang Gung Memorial Hospital Taipei Branch, Taipei 10507, Taiwan; lin0807@cgmh.org.tw; 4 Graduate Institute of Clinical Medical Sciences, Chang Gung University, Taoyuan 33302, Taiwan; 5 Institute of Emergency and Critical Care Medicine, National Yang Ming Chiao Tung University, Taipei 112304, Taiwan

**Keywords:** cerebral blood flow, cerebral hemodynamics, Doppler ultrasonography, extremely preterm infants, near-infrared spectroscopy (NIRS)

## Abstract

**Highlights:**

**What are the main findings?**

• Longitudinal reference values for middle cerebral artery blood flow velocity were established in extremely preterm infants using multimodal criteria for physiological stability.
• Postmenstrual age and concurrent body weight were independently associated with middle cerebral artery blood flow velocity, whereas mean blood pressure was not.

**What are the implications of the main findings?**

• These longitudinal reference values provide a physiological framework for interpreting cerebral blood flow velocity throughout postnatal maturation in preterm infants.
• Multimodal hemodynamic assessment, including regional cerebral tissue oxygenation, may improve the clinical evaluation of adequate cerebral perfusion beyond the use of blood pressure alone.

**Abstract:**

Background: Cerebral blood flow (CBF) is essential for maintaining cerebral metabolism in extremely preterm infants; however, no universally accepted reference standard exists for CBF velocity (CBFV). This study aimed to establish physiological reference patterns of middle cerebral artery CBFV under strictly defined physiological stability using multimodal monitoring. Methods: This post-hoc analysis included extremely preterm infants (gestational age ≤ 28^+6^ weeks or birthweight 500–1000 g) from a prospective cohort study. Serial Doppler ultrasonography of the middle cerebral artery was performed along with near-infrared spectroscopy and electrical cardiometry. Normal CBFV datasets were defined based on systemic stability, adequate cardiac output (>150 mL/kg/min), normal regional cerebral oxygen saturation (65–85%), and the absence of major comorbidities. Associations between Doppler parameters and postmenstrual age (PMA) and concurrent weight were analyzed using generalized estimating equations. Results: Forty infants contributed 194 normal CBFV datasets. Physiological reference ranges for peak systolic velocity (PSV), end-diastolic velocity (EDV), mean velocity (MV), resistance index (RI), and pulsatility index (PI) were established across PMA and concurrent weight strata. Both PSV and MV were associated with PMA and concurrent weight (*p* < 0.01), whereas EDV, RI, and PI were not. Mean blood pressure was not significantly associated with CBFV parameters. Conclusions: PSV and MV were maturation-dependent cerebral perfusion markers. Multimodally defined physiological stability provides a robust framework for establishing clinically relevant reference values for middle cerebral artery CBFV in extremely preterm infants.

## 1. Introduction

Cerebral blood flow (CBF) is a fundamental component of cerebral hemodynamics that ensures adequate delivery of oxygen and nutrients required for normal brain function. Maintaining stable and appropriate CBF in extremely preterm infants is critical because their structurally fragile cerebral vasculature and immature autoregulatory capacity render them highly vulnerable to neurological injuries. Both cerebral hypoperfusion, which may result in ischemic damage and hyperperfusion, which increases the risk for hemorrhage, should be avoided in this population [1].

Fluctuations in cerebral perfusion have been consistently identified as major contributors to intraventricular hemorrhage (IVH) and white matter injury in preterm infants [2,3,4]. Although stabilization of systemic blood pressure (BP) has traditionally been a central strategy for reducing the risk for IVH, emerging evidence suggests that BP alone may not adequately reflect cerebral perfusion or predict long-term neurological outcomes [5,6]. These findings underscore the complexity of cerebral hemodynamics, in which systemic parameters represent only one component of a multifaceted regulatory system.

In addition to postnatal challenges, cerebral perfusion must also be considered in the context of physiological development. During gestation, CBF increases progressively in parallel with rapid brain growth and rising metabolic demand, supporting key processes, such as neurogenesis and neuronal migration [7]. Accordingly, normative reference ranges for fetal CBF have been established using Doppler ultrasonography and are routinely applied in the surveillance of high-risk pregnancies [8,9,10].

However, only a limited number of studies have attempted to define normative CBF ranges in preterm infants, largely due to the significant influence of physiological and pathological variables on cerebral perfusion [11,12,13]. The widely cited reference values reported by Romagnoli et al. [12] were established under clinical conditions that differed substantially from those in contemporary neonatal care. Advances in respiratory management, carbon dioxide targeting, oxygen saturation strategies, and metabolic support have fundamentally altered the hemodynamic milieu of preterm infants. Therefore, an updated CBF reference range, based on modern clinical practice, is necessary.

In contrast, there is no universally accepted definition of normal CBF in extremely preterm infants. Previous studies have reported reference values for CBF velocities. However, these values have often been derived from heterogeneous populations and variable definitions of physiological stability [11,12,14,15]. As a result, gestational age (GA)-categorized or condition-specific reference ranges remain poorly defined, limiting clinicians’ ability to accurately interpret cerebral hemodynamic status and to detect early deviations from optimal perfusion.

Recent advances in non-invasive multimodal monitoring, however, have offered new opportunities to address this gap. Technologies, such as near-infrared spectroscopy (NIRS) for regional cerebral oxygen saturation (rcSO_2_), and electrical cardiometry (EC) for non-invasive cardiac output (CO) measurement, enable continuous bedside assessment of cerebral and systemic hemodynamics [16,17]. NIRS is used to estimate rcSO_2_ based on the differential absorption of near-infrared light by oxygenated and deoxygenated hemoglobin, and is widely used for cerebral oxygenation monitoring in preterm infants. EC provides non-invasive CO assessment based on thoracic bioimpedance and has been validated in preterm populations [18,19]. Integration of these modalities enables a more comprehensive and dynamic evaluation of cerebral perfusion beyond static measurements. Although pulsed-wave Doppler ultrasonography measures CBF velocity (CBFV) rather than absolute volumetric blood flow, direct quantification of CBF requires accurate measurement of the vessel diameter, which is not routinely feasible in clinical practice, particularly in extremely preterm infants. Therefore, Doppler-derived CBFV serves as a practical and widely accepted surrogate for serial bedside assessments of cerebral perfusion.

The present study aimed to define longitudinal physiological reference values for CBFV in extremely preterm infants under strictly controlled physiological conditions. By performing Doppler ultrasonography with multimodal cerebral hemodynamic monitoring, this study sought to provide a practical physiological reference for bedside assessment of cerebral perfusion in this vulnerable population.

## 2. Materials and Methods

### 2.1. Study Population

The present study was a post-hoc secondary analysis of a prospective observational cohort investigating multimodal hemodynamic monitoring in extremely preterm infants with GA ≤ 28^+6^ weeks or birth weight (BW) of 500–1000 g [20].

According to the original study design, infants were excluded if they had major congenital anomalies, evidence of perinatal asphyxia, required chest compressions resuscitation, or had IVH grade ≥ 2 before enrollment. The study was approved by the Institutional Review Board (202102305B0C601) of Chang Gung Memorial Hospital, and written informed consent was obtained from the parents.

### 2.2. Cranial Ultrasonography

According to the protocol, cranial ultrasonography examinations were performed daily during the first 3 days of life, weekly until 4 weeks of age, and monthly thereafter until discharge or term-equivalent age. All ultrasonographic examinations were performed by one of 3 neonatologists (W.H.W, S.Y.L., and K.H.H.), who were experienced members of the neonatal functional echography team.

**Figure 1:**
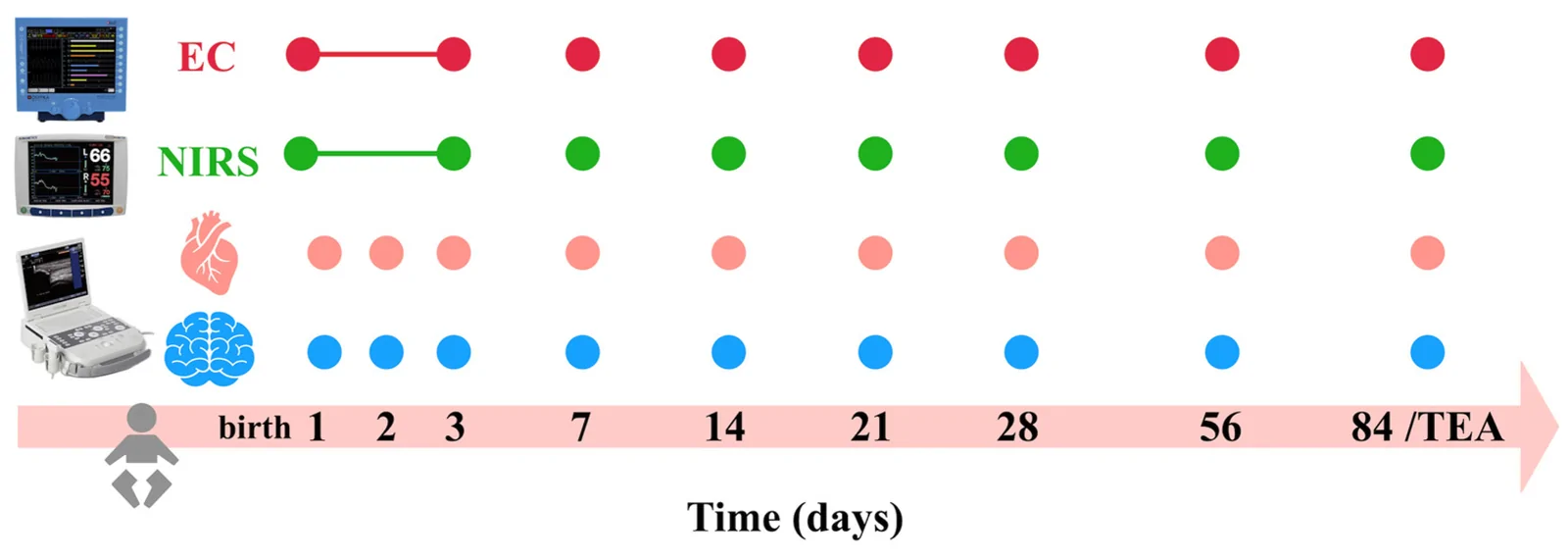
Study protocol for serial multimodal hemodynamic monitoring in preterm infants. Measurements were obtained at birth and on postnatal days 1, 2, 3, 7, 14, 21, 28, 56, and 84 (term-equivalent age, TEA). Electrical cardiometry (EC) and near-infrared spectroscopy (NIRS) were performed longitudinally at all time points. Functional echocardiography was used for cardiac assessment, and cranial Doppler ultrasound was performed to evaluate cerebral blood flow parameters.

**Figure A1:**
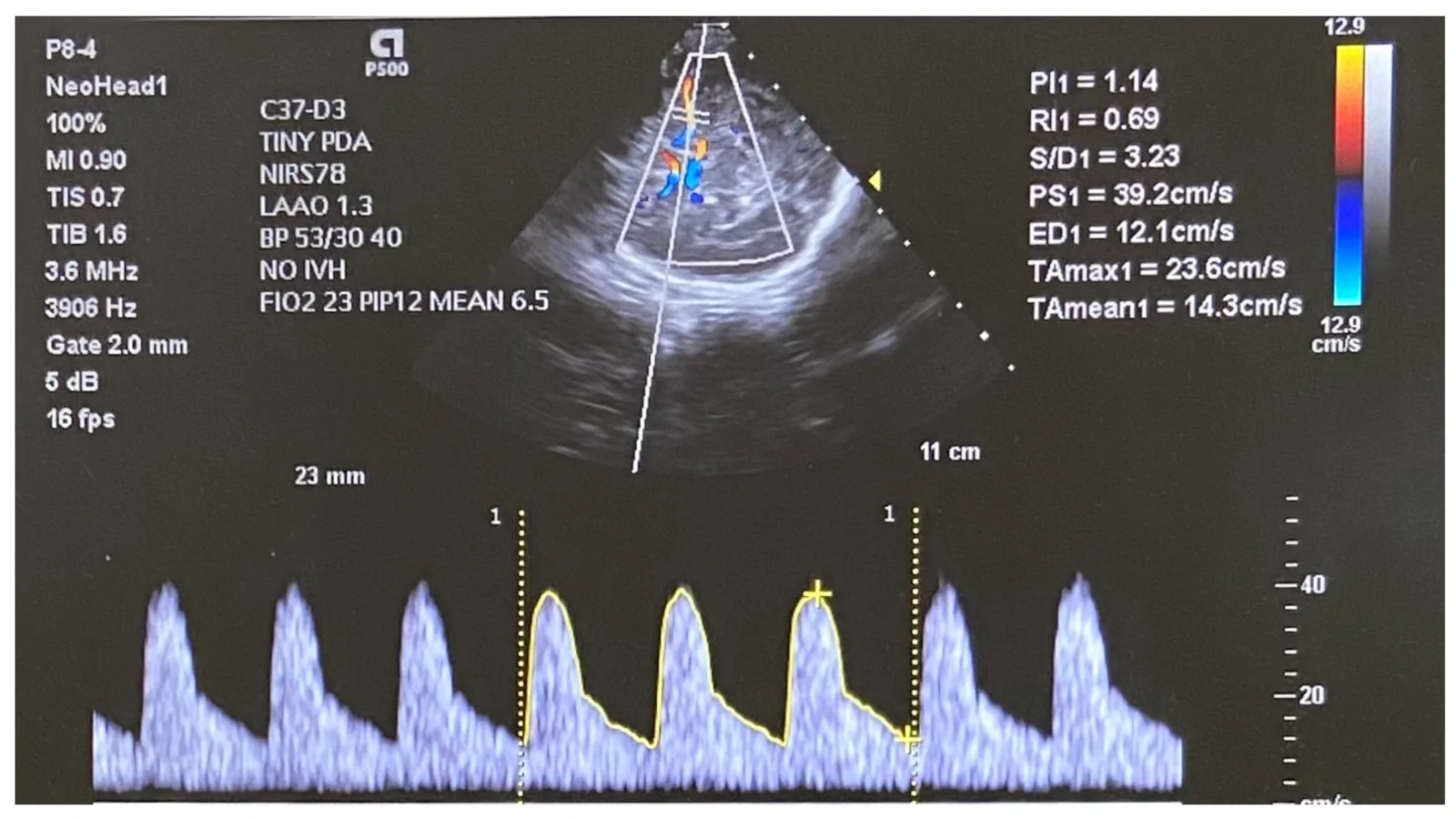
Representative middle cerebral artery (MCA) Doppler waveform obtained using cranial ultrasound in a preterm infant. TAMAX (time-averaged maximum velocity) is referred to as mean velocity (MV) throughout this manuscript.

The study protocol is illustrated in Figure 1. The infants were maintained in the supine position and examined in a calm state with minimal handling or non-nutritive sucking. Examinations were performed using an ultrasound system (Acuson P500, Siemens Healthcare, Erlangen, Germany) equipped with a 4–11 MHz transducer. Examinations focused on IVH and CBFV assessments. Doppler measurements were obtained from the left middle cerebral artery (MCA), which has been widely used as a surrogate for cerebral perfusion in fetal and neonatal populations. Doppler velocity parameters included peak systolic velocity (PSV), end-diastolic velocity (EDV), and mean velocity (MV). MV in this study refers to the time-averaged maximum velocity (TAMAX) displayed by the ultrasound system. This terminology is used throughout this article for simplicity and consistency with other Doppler-derived velocity parameters. The Doppler insonation angle was maintained as close as possible to 0° with appropriate angle correction. To ensure accuracy, measurements were recorded from stable flow waveforms over ≥10 consecutive cardiac cycles with clear spectral signals (Appendix A, Figure A1). The pulsatility index (PI; [PSV–EDV]/MV) and resistance index (RI; [PSV–EDV]/PSV) were automatically calculated by the ultrasound device.

### 2.3. Hemodynamic Monitoring

Targeted neonatal echocardiography was performed concurrently with cranial ultrasonography using the same ultrasound device and probe to assess the presence of a patent ductus arteriosus (PDA). In addition, simultaneous hemodynamic monitoring was performed using NIRS (INVOS 5100C, Medtronic, Minneapolis, MN, USA) for rcSO_2_ and EC (Aesculon, Osypka Medical, Berlin, Germany) for non-invasive CO monitoring (Figure 1). These multimodal physiological measurements were interpreted together with Doppler-derived CBFV to characterize cerebral perfusion under physiologically stable conditions, including: heart rate (HR), BP, arterial oxygen saturation (SpO_2_), rcSO_2_, and CO.

### 2.4. Definition of Physiologically Stable Cerebral Perfusion

A predefined multimodal physiological screening protocol was used to identify infants with physiologically stable cerebral perfusion before their inclusion in the reference cohort. Eligible examinations were required to fulfill the following criteria:

1. Stable HR (120–180 beats/min), BP (mean BP ≥ postmenstrual age [PMA] in weeks), SpO_2_ (≥90%), and without any inotrope(s) support;
2. Normothermia, body temperature 36.5–37.5 °C;
3. Normal metabolic status (normal blood acid–base balance and no hypoglycemia < 40 mg/dL);
4. Adequate CO (>150 mL/kg/min) [18];
5. Normal cerebral oxygenation, indicating rcSO_2_ within 65–85% and normal fractional tissue oxygen extraction (FTOE; calculated as SpO_2_ − rcSO_2_/SpO_2_) within 10–40% [21];
6. No recent red blood cell transfusion within the preceding week;
7. No evidence of systemic infection;
8. Absence of IVH grade ≥ 2 or evidence of seizure;
9. No hemodynamically significant PDA (defined as a ductal diameter > 2.0 mm or left atrium-to-aortic root ratio > 1.4) [22].

As part of routine neonatal intensive care unit practice, arterial blood gas analysis, biochemistry data, and hemograms were obtained every 6–24 h during the acute phase and weekly once the infant was stable. Doppler ultrasound examinations were scheduled within 1 h of routine blood sampling whenever feasible, enabling contemporaneous assessment of partial pressure of carbon dioxide (PaCO_2_) and hemoglobin levels before inclusion in the physiologically stable cohort. Only observations that fulfilled all predefined physiological stability criteria were included in the final analysis. Observations that did not fulfill these criteria were excluded at the observation level, whereas the corresponding infants remained eligible for subsequent analyses if later examinations fulfilled the inclusion criteria.

### 2.5. Statistics

Associations between Doppler-derived MCA velocity parameters (i.e., PSV, EDV, MV, PI, and RI) were evaluated using generalized estimating equations (GEE). Multivariate GEE models were constructed for each Doppler parameter. PMA or concurrent weight was entered as the primary explanatory variable in separate models, whereas the measurement sequence and mean BP were included as adjustment covariates to account for the potential temporal effects of serial measurements and the known influence of systemic BP on cerebral perfusion. An independent working correlation structure with robust sandwich standard errors was selected because the number and timing of eligible observations varied among the infants, resulting in an unbalanced longitudinal dataset. This approach accounted for the clustering of repeated observations within the same infant while permitting unequal numbers of measurements. Only observations fulfilling all predefined physiological stability criteria were included, and excluded or unavailable measurements were not imputed. Regression coefficient (β), 95% confidence interval, and corresponding *p* value are reported. Differences with *p* < 0.05 were considered to be statistically significant.

## 3. Results

**Figure 2:**
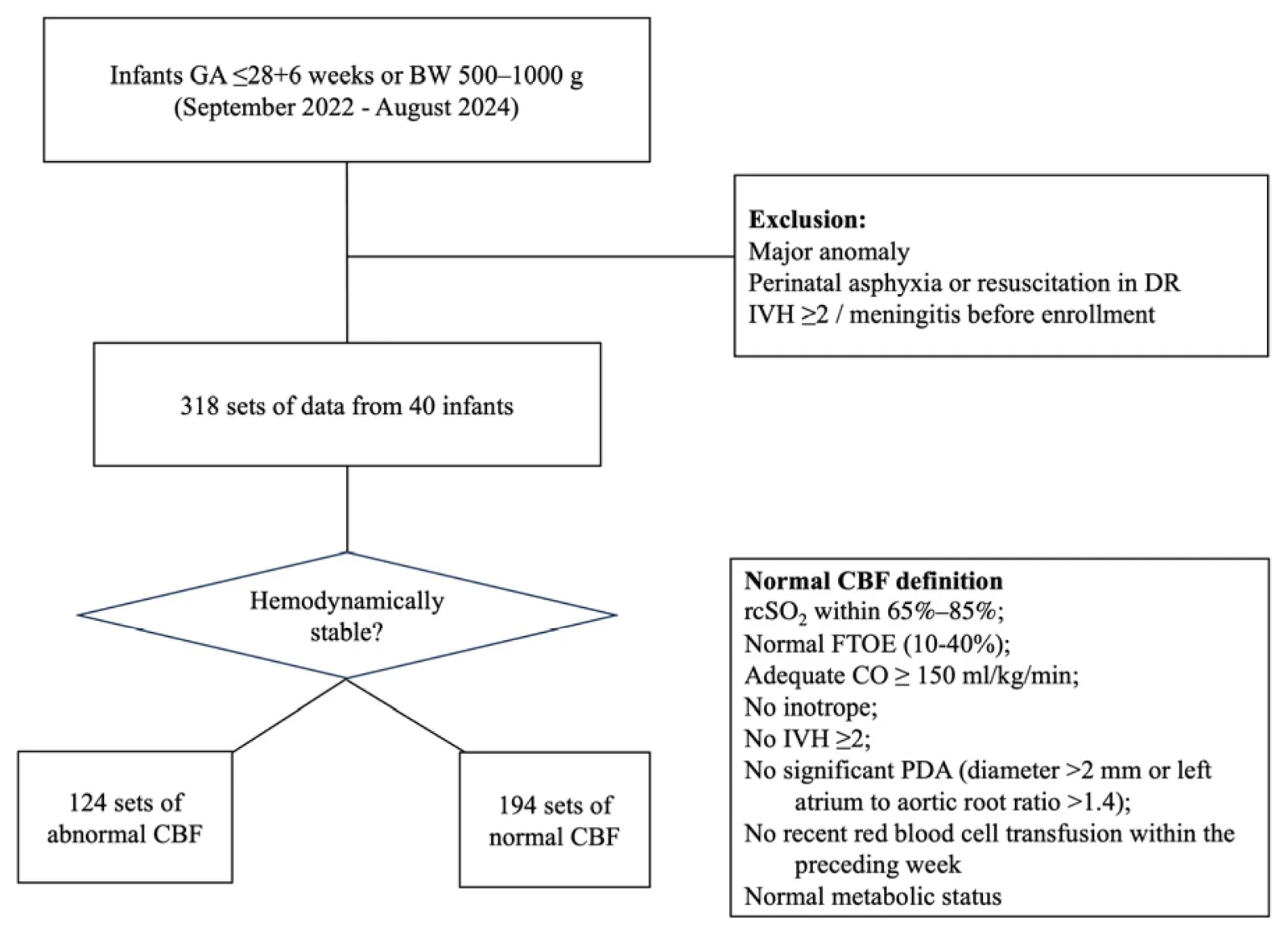
Flow diagram of patient enrollment and the definition of normal CBF.

Data from 40 extremely preterm infants, collected between September 2022 and August 2024, were analyzed. Overall, 318 datasets were collected, of which 194 fulfilled the predefined criteria for normal CBF, with a median of 5 examinations per infant (interquartile range, 3–7; range, 1–9) (Figure 2). The mean (±SD) GA was 26.9 ± 2.0 weeks, and BW 810.0 ± 220.5 g. Seventeen (42.5%) infants were male, and 9 (22.5%) were small-for-gestational age (BW < 10th percentile). Demographic data are summarized in Table 1.

**Figure 3:**
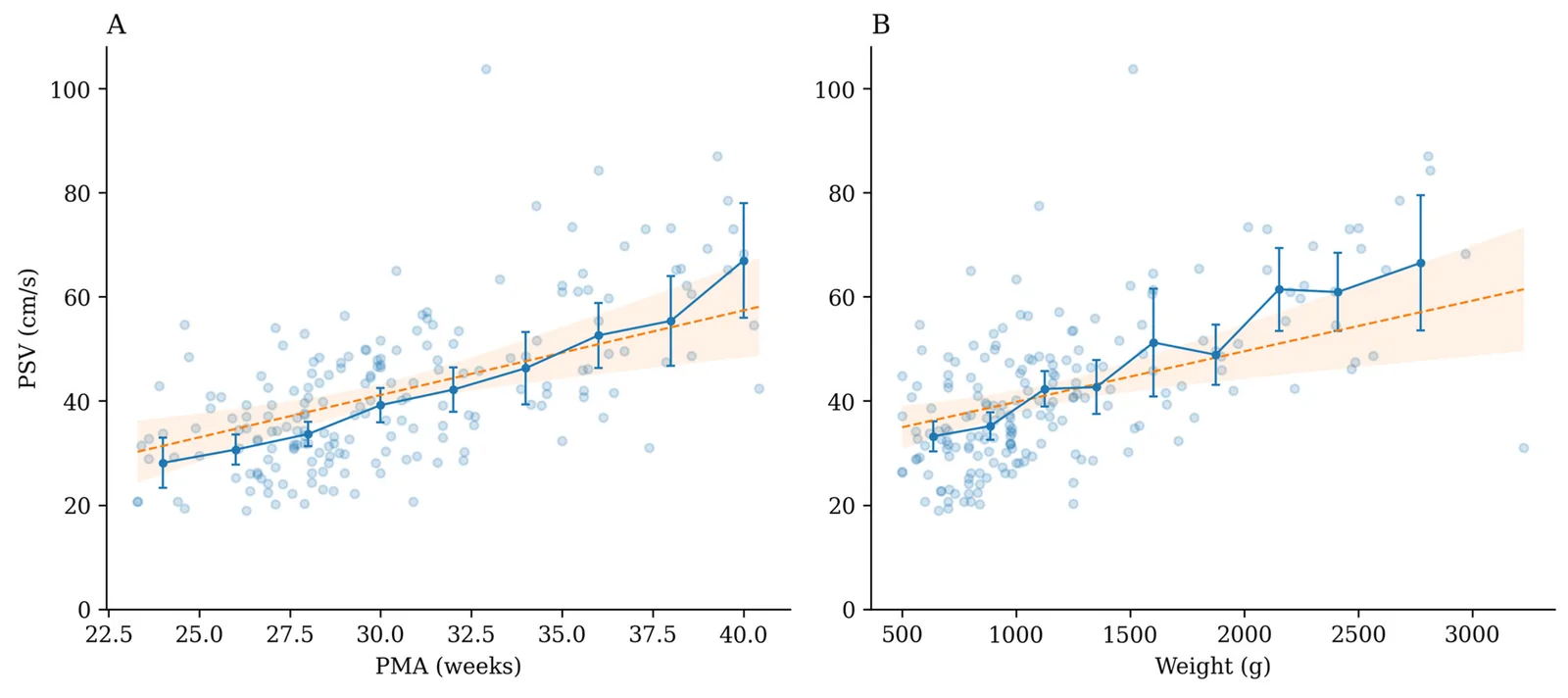
Association of middle cerebral artery peak systolic velocity (PSV) with (**A**) postmenstrual age (PMA) and (**B**) concurrent weight. Points represent individual observations. Solid lines with error bars denote group means with 95% confidence intervals. Dashed lines and shaded areas represent GEE model-estimated trends and 95% confidence intervals.

The distributions of the longitudinal physiological reference values of the Doppler parameters stratified according to PMA and concurrent weight are summarized in Table 2 and Table 3. Across PMA and concurrent weight ranges, PSV ranged from 18.9 to 103.9 cm/s, and MV ranged from 11.0 to 48.5 cm/s. As illustrated in Figure 3, the PSV exhibited a progressive increase in both PMA and weight. In panel A, the PSV increased steadily with advancing PMA, whereas in panel B, a similar upward trend is apparent with increasing weight. Despite inter-individual variability, the overall distribution and regression trends consistently exhibited a positive association between the PSV and maturation parameters.

Using multivariate GEE analyses, both PMA and concurrent weight were independently associated with PSV and MV, after adjusting for mean BP and predefined follow-up time points. PMA was positively associated with PSV (β = 1.45, 95% CI 0.42–2.47; *p* < 0.01) and MV (β = 0.62, 95% CI 0.06–1.17; *p* = 0.02). Similarly, concurrent weight (per 100 g increase) was positively associated with PSV (β = 1.00, 95% CI 0.42–1.53; *p* < 0.01) and MV (β = 0.40, 95% CI 0.11–0.71; *p* < 0.01). In contrast, EDV, PI, and RI were not significantly associated with PMA or concurrent weight (all *p* > 0.05) (Appendix A, Table A1).

## 4. Discussion

This is the first study to integrate NIRS and EC, along with conventional hemodynamic parameters and clinical status, to define physiological stability and cerebral metabolic conditions. This multimodal approach enables a more rigorous selection of stable states for the assessment of CBFV, thereby enhancing the interpretability and clinical relevance of Doppler-derived measurements. To our knowledge, this is the first study to characterize the relationship between CBFV and maturation using longitudinal data from a cohort of extremely preterm infants.

Traditionally, Doppler-derived indices, such as RI, have been used as indirect markers of CBF, in part, because they are less affected by the angle of insonation. However, previous studies have demonstrated a poor correlation between RI and CBF, particularly during the early postnatal period [23]. In contrast, our findings indicated that PSV and MV demonstrated stronger associations with PMA and concurrent weight, which is consistent with previous reports [12,14,23]. The observed increase in PSV and MV with advancing age and weight likely reflects the progressive maturation of the cerebral vascular bed and increased cerebral blood volume. Physiologically, PSV represents the peak systolic flow driven by CO, whereas MV provides a more integrated measure of perfusion across the cardiac cycle. Therefore, these parameters may better capture the developmental trajectory of the cerebral circulation than resistance-based indices. From a clinical perspective, these findings support the use of velocity-based measurements as sensitive markers for monitoring cerebral perfusion and guiding neuroprotective strategies [24].

In contrast, the PI and RI exhibited inconsistent correlations with the PMA or concurrent weight in our cohort. Although these indices are widely used to assess cerebrovascular resistance and intracranial dynamics, their physiological interpretation in preterm infants remains complex. Cerebral vascular resistance is influenced by multiple factors including acid–base status, oxygenation, PaCO_2_ levels, and cerebral perfusion pressure [25,26]. Given that the neonatal cerebral circulation is inherently a low-resistance system, variations in PI and RI may reflect transient physiological fluctuations rather than stable developmental changes. Moreover, previous studies have reported inconsistent relationships between vascular resistance indices and maturation [27]. Collectively, our findings suggest that the reliance on RI or PI alone is insufficient to assess CBF in extremely preterm infants, reinforcing the need for multimodal and velocity-based approaches.

The establishment of normative CBFV values provides an important physiological benchmark for evaluating cerebral hemodynamics and systemic perfusion in critically ill preterm infants. Although BP remains the primary focus in neonatal intensive care, it does not necessarily reflect cerebral perfusion or predict neurological outcomes. By incorporating multimodal physiological stability criteria, the present study minimized the influence of transient systemic and cerebral hemodynamic disturbances on Doppler measurements and may therefore provide physiologically more robust reference values.

A comparison with previous neonatal reference studies (Appendix A, Table A2) demonstrated that the developmental trends of MCA Doppler parameters observed in the present study were generally consistent with those reported by Pezzati et al., Romagnoli et al., and Forster et al. [11,12,14]. Nevertheless, a direct comparison of absolute Doppler values should be interpreted cautiously because of differences in study populations, measurement timing, ultrasound acquisition protocols, and definitions of physiological normality. Consequently, the proposed longitudinal reference values may provide a more robust physiological framework for interpreting CBFV during postnatal maturation in extremely preterm infants. Measurements outside these physiological reference ranges should not be regarded as diagnostic of a specific pathological condition but rather as an indication for further clinical evaluation. Depending on the overall clinical context, abnormal Doppler findings may prompt the consideration of conditions known to influence cerebral hemodynamics, including alterations in PaCO_2_, severe anemia, impaired cerebral autoregulation, significant PDA, and other causes of cerebral hypoperfusion or hyperperfusion [28]. Similarly, deviations in EDV, MV, PI, or RI may reflect changes in cerebrovascular resistance or downstream perfusion, and should be interpreted together with other clinical, echocardiographic, and hemodynamic findings rather than in isolation [22,29,30,31,32]. Early recognition of Doppler measurements that fall outside the expected physiological range may facilitate timely assessment of potentially reversible disturbances in cerebral perfusion. Nevertheless, the present study was designed to establish longitudinal physiological reference values rather than to evaluate pathological cerebral hemodynamics or neurological outcomes. Therefore, the application of these reference values for diagnosing specific disease states or predicting brain injury should be considered hypothesis-generating and requires validation in future prospective studies involving infants with abnormal cerebral hemodynamics.

The present study had several limitations. First, the sample size was relatively small (i.e., 40 infants and 194 qualified datasets), which may have limited the statistical power and the ability to capture the full spectrum of physiological variability. Second, the single-center design may have reduced the generalizability of the findings to other populations and clinical settings. Multicenter studies with larger cohorts are required to validate and refine these reference values. Third, stringent inclusion criteria, which are essential for internal validity, may limit clinical applicability. After excluding infants requiring inotropic support, those with hemodynamic instability, and those with metabolic disturbances, the study population represented a highly selected cohort of physiologically stable infants. This approach strengthens interpretability but may not reflect the complexity of routine neonatal intensive care. Fourth, some hemodynamic threshold may be oversimplified. For example, a fixed rcSO_2_ range (65–85%) was used as part of the physiological stability criterion in this study. Although this threshold has been widely adopted in neonatal monitoring to identify clinically acceptable cerebral oxygenation, recent evidence suggests that rcSO_2_ varies according to GA, postnatal age, and the NIRS device or sensor used [33]. Future studies that incorporate GA and postnatal age-specific centile charts may further optimize the definition of normal cerebral hemodynamics. Likewise, the diagnosis of hsPDA generally requires additional echocardiographic evidence of significant left-to-right shunting and systemic steal beyond ductal size or the left atrium-to-aortic root ratio alone. Therefore, these thresholds should be interpreted cautiously and individualized according to the clinical context.

## 5. Conclusions

The present study established age- and weight-dependent reference patterns for CBFV in extremely preterm infants under rigorously defined physiological stability and multimodal monitoring conditions. Velocity-based Doppler parameters, particularly PSV and MV, demonstrated stronger activation with maturation than resistance indices, supporting their utility as sensitive markers of cerebral perfusion. Our findings provide a clinically relevant framework for interpreting cerebral hemodynamics and may facilitate earlier identification of abnormal cerebral perfusion.

## Figures and Tables

**Table 1:** Demographics of the study population.

Demographics	*n* = 40
Gestation age (weeks)	26.9 ± 2.0
Birth weight (g)	810.0 ± 220.5
Male	17 (42.5%)
Small-for-gestational age	9 (22.5%)
Apgar score at 1 min	6 ± 1
Apgar score at 5 min	8 ± 1
Antenatal steroids	35 (87.5%)
Antenatal magnesium sulfate	30 (75%)
Bacteremia	11 (27.5%)
Ligation for patent ductus arteriosus	11 (27.5%)
Spontaneous intestinal perforation	4 (10%)
Necrotizing enterocolitis (any stage)	0 (0%)
Intraventricular hemorrhage ≥ 2	2 (5%)
Retinopathy of prematurity (any stage)	4 (10%)
Mortality	5 (12.5%)

Data are mean ± standard deviation or *n* (%).

**Table 2:** Longitudinal physiological reference values of Doppler parameters stratified by PMA.

PMA (Weeks)	≤24	25–26	27–28	29–30	31–32	33–34	35–36	37–38	≥39
Datasets (*n*)	16	22	51	35	24	11	17	10	8
PSV (cm/s)	29.2 ± 10.1	31.4 ± 7.1	33.1 ± 7.2	40.1 ± 10.0	45.5 ± 10.1	45.6 ± 12.3	54.7 ± 14.0	62.2 ± 13.8	68.8 ± 13.8
MV (cm/s)	18.1 ± 3.8	18.2 ±3.8	18.2 ± 4.2	21.3 ± 5.8	23 ± 7.8	24.8 ± 7.9	27.6 ± 6.6	33.4 ± 8.6	33.2 ± 7.2
EDV (cm/s)	8.9 ± 2.8	9.3 ± 2.3	9.5 ± 3.0	9.7 ± 3.6	10.8 ± 3.1	11.7 ± 4.6	12.3 ± 3.4	13 ± 5.5	15.8 ± 3.3
PI	1.2 ± 0.2	1.2 ± 0.2	1.3 ± 0.3	1.4 ± 0.3	1.4 ± 0.3	1.4 ± 0.2	1.5 ± 0.2	1.5 ± 0.2	1.5 ± 0.2
RI	0.7 ± 0.1	0.7 ± 0.1	0.7 ± 0.1	0.8 ± 0.1	0.8 ± 0.1	0.7 ± 0.1	0.8 ± 0.1	0.7 ± 0.1	0.8 ± 0.1

**Table 3:** Longitudinal physiological reference values of Doppler parameters for distinct concurrent weight groups.

Weight (g)	≤750	751–1000	1001–1250	1251–1500	1501–1750	1751–2000	2001–2250	2251–2500	≥2501
Datasets (*n*)	38	56	36	16	15	7	6	8	8
PSV (cm/s)	31.4 ± 9.3	33.1 ± 10	42.1 ± 11.3	43.5 ± 9.4	45.8 ± 19.1	49.1 ± 8.4	60.9 ± 10.7	61.6 ± 10.8	68.8 ± 18.8
MV (cm/s)	18 ± 5.3	18.9 ± 5.5	21.6 ± 6.6	21.7 ± 3.7	25 ± 8.8	25.7 ± 6.8	30.4 ± 5.7	33.4 ± 5.6	37.1 ± 9.6
EDV (cm/s)	8.7 ± 3.1	9.2 ± 3.3	10.8 ± 3.7	10.2 ± 2.3	10.8 ± 3.2	13.4 ± 4.6	11.8 ± 2.6	13.0 ± 3.7	16.1 ± 5.3
PI	1.3 ± 0.2	1.3 ± 0.3	1.4 ± 0.3	1.5 ± 0.3	1.4 ± 0.4	1.3 ± 0.3	1.6 ± 0.2	1.5 ± 0.2	1.5 ± 0.2
RI	0.7 ± 0.1	0.7 ± 0.1	0.7 ± 0.1	0.8 ± 0.1	0.8 ± 0.1	0.7 ± 0.1	0.8 ± 0.1	0.8 ± 0.1	0.8 ± 0.1

## Data Availability

Raw data supporting the findings of this study are available from the corresponding author upon reasonable request. The data are not publicly available due to patients’ privacy.

## Appendix A

**Table A1:** Multivariable GEE analyses of middle cerebral artery blood flow velocity parameters according to postmenstrual age and concurrent weight. Separate multivariable generalized estimating equation models were constructed using either postmenstrual age or concurrent weight as the primary explanatory variable. Both models were adjusted for mean BP and predefined follow-up time point. An independence working correlation structure with robust sandwich standard errors was used. β represents the adjusted change in each Doppler-derived parameter per 1-week increase in PMA or per 100 g increase in concurrent weight.

	PMA β (95% CI)	*p* Value	Concurrent Weight (per 100 g), β (95% CI)	*p* Value
PSV, cm/s	1.45 (0.42–2.47)	<0.01 *	1.01 (0.42–1.53)	<0.01 *
MV, cm/s	0.62 (0.06 -1.17)	0.02 *	0.41 (0.11–0.71)	<0.01 *
EDV, cm/s	0.26 (−0.05–0.57)	0.11	0,01 (−0.01–0.03)	0.29
PI	0.02 (−0.01–0.04)	0.17	0.01 (−0.01–0.03)	0.07
RI	0.01 (−0.01–0.01)	0.08	0.01 (−0.01–0.01)	0.14

PSV: peak systolic velocity; EDV: end-diastolic velocity; MV: mean velocity; PI: pulsatility index; RI: resistance index. * indicates significant *p*-value < 0.05 based on generalized estimating equation for longitudinal data.

**Table A2:** Comparison of Published Reference Studies of Middle Cerebral Artery Doppler Parameters in Neonates.

Study	Population	Sample Size	Definition of “Normal”	MCA Doppler Values ^†^
Pezzati et al. [11]	Healthy preterm and term infants (GA 24–41 weeks)	120 infants (120 examinations)	Appropriate-for-gestational-age neonates without congenital anomalies, birth asphyxia, respiratory distress syndrome, patent ductus arteriosus, intracranial hemorrhage, metabolic disturbances, abnormal blood gases, or cardiovascular disease; examined 2–8 h after birth.	24–28 weeks: PSV 20 ± 6 cm/s, EDV 4.7 ± 2.0 cm/s, MV 10 ± 3 cm/s, RI 0.76 ± 0.07 29–32 weeks: PSV 26 ± 7 cm/s, EDV 5.0 ± 2.0 cm/s, MV 13 ± 4 cm/s, RI 0.80 ± 0.07 33–37 weeks: PSV 29 ± 9 cm/s, EDV 5.1 ± 2.0 cm/s, MV 14 ± 5 cm/s, RI 0.82 ± 0.07 38–41 weeks: PSV 31 ± 8 cm/s, EDV 5.7 ± 2.0 cm/s, MV 16 ± 4 cm/s, RI 0.81 ± 0.08
Romagnoli et al. [12]	Healthy preterm infants (GA 25–35 weeks)	70 infants (732 examinations)	Strict clinical stability: no fetal distress, chorioamnionitis, congenital anomaly, abnormal cranial ultrasound, significant PDA, hypotension, abnormal blood gases, abnormal hematocrit, or medications affecting cerebral circulation; measurements obtained after 12 h of life.	25–28 weeks: PSV 21.0 cm/s, MV 12.0 cm/s, EDV 6.0 cm/s, PI 1.37, RI 0.75 29–30 weeks: PSV 27.2 cm/s, MV 14.2 cm/s, EDV 7.2 cm/s, PI 1.27, RI 0.73 31–32 weeks: PSV 28.8 cm/s, MV 15.5 cm/s, EDV 8.0 cm/s, PI 1.28, RI 0.73 33–35 weeks: PSV 31.8 cm/s, MV 18.2 cm/s, EDV 8.0 cm/s, PI 1.35, RI 0.74
Present study	Extremely preterm infants (GA ≤ 28 weeks or BW 500–1000 g)	40 infants (194 physiologically stable datasets from 318 examinations)	Multimodal physiological stability: cardiac output > 150 mL/kg/min, rcSO_2_ 65–85%, no hsPDA No systemic fluid imbalance, and no major neonatal morbidities	PMA ≤ 24 weeks: PSV 29.2 ± 10.1 cm/s, MV 18.1 ± 3.8 cm/s, EDV 8.9 ± 2.8 cm/s, PI 1.2 ± 0.2, RI 0.7 ± 0.1 PMA 25–26 weeks: PSV 31.4 ± 7.1 cm/s, MV 18.2 ± 3.8 cm/s, EDV 9.3 ± 2.3 cm/s, PI 1.2 ± 0.2, RI 0.7 ± 0.1 PMA 27–28 weeks: PSV 33.1 ± 7.2 cm/s, MV 18.2 ± 4.2 cm/s, EDV 9.5 ± 3.0 cm/s, PI 1.3 ± 0.3, RI 0.7 ± 0.1 PMA 29–30 weeks: PSV 40.1 ± 10.0 cm/s, MV 21.3 ± 5.8 cm/s, EDV 9.7 ± 3.6 cm/s, PI 1.4 ± 0.3, RI 0.8 ± 0.1 PMA 31–32 weeks: PSV 45.5 ± 10.1 cm/s, MV 23.0 ± 7.8 cm/s, EDV 10.8 ± 3.1 cm/s, PI 1.4 ± 0.3, RI 0.8 ± 0.1 PMA 33–34 weeks: PSV 45.6 ± 12.3 cm/s, MV 24.8 ± 7.9 cm/s, EDV 11.7 ± 4.6 cm/s, PI 1.4 ± 0.2, RI 0.7 ± 0.1 PMA 35–36 weeks: PSV 54.7 ± 14.0 cm/s, MV 27.6 ± 6.6 cm/s, EDV 12.3 ± 3.4 cm/s, PI 1.5 ± 0.2, RI 0.8 ± 0.1 PMA 37–38 weeks: PSV 62.2 ± 13.8 cm/s, MV 33.4 ± 8.6 cm/s, EDV 13.0 ± 5.5 cm/s, PI 1.5 ± 0.2, RI 0.7 ± 0.1 PMA ≥ 39 weeks: PSV 68.8 ± 13.8 cm/s, MV 33.2 ± 7.2 cm/s, EDV 15.8 ± 3.3 cm/s, PI 1.5 ± 0.2, RI 0.8 ± 0.1
Forster et al. [14]	Healthy term neonates	38 infants (38 examinations)	Healthy term neonates without cardiorespiratory or neurological disease; examined during the first 72 h after birth	PSV: 41.4 ± 13.2 cm/s MV: 25.8 ± 7.9 cm/s EDV: 13.0 ± 5.5 cm/s PI: 1.17 ± 0.23 RI: 0.68 ± 0.07

GA, gestational age; PMA, postmenstrual age; BW, birth weight; MCA, middle cerebral artery; PSV, peak systolic velocity; EDV, end-diastolic velocity; MV, mean velocity (time-averaged maximum velocity, TAMAX); PI, pulsatility index; RI, resistance index; PDA, patent ductus arteriosus; rcSO_2_, regional cerebral oxygen saturation. **^†^** Representative Doppler values were extracted from the vessel evaluated in each original study (Pezzati et al.: right MCA; Romagnoli et al.: MCA; Forster et al.: MCA; present study: left MCA). Values are presented as mean ± standard deviation unless otherwise specified. Romagnoli et al. reported the 50th percentile (median) values for each gestational-age group.
